# Advancing the pain management in older adults agenda forward through the development of key research and education priorities: A Canadian perspective

**DOI:** 10.1080/24740527.2017.1383139

**Published:** 2017-10-26

**Authors:** Sharon Kaasalainen, Ramesh Zacharias, Courtney Hill, Abigail Wickson-Griffiths, Thomas Hadjistavropoulos, Keela Herr

**Affiliations:** a Department of Family Medicine, School of Nursing, McMaster University, Hamilton, Ontario, Canada; b Chronic Pain Management Unit, Chedoke Hospital & Village of Erin Meadows, Hamilton, Ontario, Canada; c School of Nursing, McMaster University, Hamilton, Ontario, Canada; d Faculty of Nursing, University of Regina, Regina, Saskatchewan, Canada; e Department of Psychology, University of Regina, Regina, Saskatchewan, Canada; f College of Nursing, University of Iowa, Iowa City, Iowa, USA

**Keywords:** Pain management, older adults, education, research

## Abstract

**Background**: The undermanagement of pain in older adults has been identified as a problem worldwide.

**Aims**: The purpose of this research is to identify priority areas in education and research for future development with the aim of improving pain management in older persons. In addition, barriers to addressing these priorities are identified.

**Methods**: This mixed methods study, based on a modified Delphi approach, included three distinct components: (1) a qualitative component using focus groups with key informants or experts in the field of pain management in older adults (*n* = 17), (2) a scoping review of the literature, and (3) a survey of ranked responses completed by the same key informants who attended the focus groups. Thematic analysis was used to identify the initial list of issues and descriptive statistics were used for ranking them.

**Results**: A number of concerns related to both education and research were frequently endorsed by participants. For education, they identified the need for more content in both undergraduate and continuing education programs related to documenting about pain; assessing pain, and learning about the complexities of pain. Research priorities included the need to explore successful practice models; costs of untreated pain; effects of mobility on pain; and patient preferences for pain management. Key barriers to addressing these barriers included lack of staff time and resources and unfamiliarity with pain assessment tools.

**Conclusion**: These findings highlight priority issues related to pain management in older adults from a nationwide perspective.

## RÉSUMÉ

**Contexte** : La prise en charge insuffisante de la douleur chez les adultes plus âgés est considérée comme un problème partout dans le monde.

**But** : Le but de cette étude est de définir les priorités en matière d’éducation et de recherche pour l’avenir, dans le but d’améliorer la gestion de la douleur chez les personnes plus âgées. De plus, les barrières existantes pour aborder ces priorités sont répertoriées.

**Méthodes** : Cette étude à méthodologie mixte, fondée sur un processus Delphi modifié, comprenait trois composantes distinctes : (1) une composante qualitative ayant recours à des groupes de discussion réunissant des informateurs clés ou des experts du domaine de la gestion de la douleur chez les adultes plus âgés (n = 17); (2) une revue exploratoire de la littérature, et (3) un sondage comprenant des questions à réponses multiples hiérarchisées à laquelle ont répondu les mêmes informateurs qui avaient participé aux groupes de discussion. Une analyse thématique a ensuite été utilisée pour dresser la liste initiale de problèmes, qui ont ensuite été classés de manière hiérarchique à l’aide de statistiques descriptives.

**Résultats** : Certaines préoccupations liées à la fois à l’éducation et à la recherche ont souvent été mentionnées par les participants. Pour l’éducation, ils ont relevé le besoin d’inclure davantage de contenu portant sur la documentation de la douleur, l’évaluation de la douleur et l’apprentissage des complexités de la douleur, tant dans les programmes de premier cycle que dans les programmes d’éducation permanente. Les priorités de recherche comprenaient la nécessité d’étudier les modèles de pratique qui ont connu du succès; les coûts de la douleur non traitée; les effets de la mobilité sur la douleur ; et les préférences des patients en matière de gestion de la douleur. Les barrières clés pour aborder ces questions comprenaient : le manque de personnel, de temps et de ressources, ainsi que la méconnaissance des outils d’évaluation de la douleur.

**Conclusion** : Ces résultats mettent en relief les questions prioritaires liées à la gestion de la douleur chez les adultes plus âgés, dans une perspective nationale.

## Introduction

The population continues to age with those aged 80 and more, representing the fastest growing segment of the population.^1^ In 2009 there were roughly 1.3 million people aged 80 or over, and this may increase to 3.3 million by 2036.^1^ With this aging population, pain management associated with many chronic health conditions will likely become an important focus of care.^2^ However, national attention needs to be focused on developing and evaluating innovative strategies to address pain management in this vulnerable population.

Numerous studies have shown that 30%–83% of older adults experience pain, with the highest rates in long term care (LTC).^3–7^ Patel et al. found that 71% of nursing home residents complained of pain.^3^ Despite these high rates of pain in older adults, pain is consistently undertreated.^8–10^ Untreated pain has both physical and psychological consequences, including weight loss, sleep disturbance, decreased functional abilities, deconditioning, increased falls, impaired mobility, depression, loneliness, anxiety, behavioral disturbance, and overall decreased quality of life.^10–13^ Hence, the problem of untreated pain warrants attention.

Pain management is particularly difficult to assess and treat in those older adults who have cognitive impairments.^14,15^ Residents with cognitive impairments are at risk for experiencing needless pain and suffering that can compromise their remaining abilities and result in declining quality of life. Horgas and Tsai^16^ used a correlational study to examine the use of analgesics in a sample of 339 residents from four nursing homes. They found that residents with cognitive impairment were prescribed and administered significantly less analgesic medication compared to cognitively intact elderly. Mezinskis et al. found that, in a chart review of 307 residents with cognitive impairment from 14 LTC facilities, fewer medications were ordered for residents with greater impairment.^17^ In addition, they found that the probability of receiving a pro re nata (prn or as needed) pain medication was significantly lower among residents with greater impairment in their ability to (1) make themselves understood and (2) understand others; the probability of receiving pain medication decreased with increasing levels of impairment. These findings are congruent with other research^11,18,19^ indicating that residents with cognitive impairments are particularly vulnerable to untreated pain and suffering.

It is believed that the problem of pain undertreatment in seniors with cognitive impairments is mainly due to challenges in the assessment of pain in this population.^20–23^ Indeed, pain assessment has proven to be a very difficult task for health care workers, largely as a result of the dementia-related impaired ability to communicate the subjective state of pain. As a result, the assessment of pain in older adults with cognitive impairment has become a topic of concern for both health care workers and researchers, which is evidenced by the emergence of a number of pain assessment tools for older adults in the literature over the past decade as well as systematic reviews of these tools.^11,20,24,25^

Clinician beliefs and attitudes about pain also play a role in how decisions are made about treatment options for older adults. For example, research has indicated that opioid medications are underutilized in seniors, particularly those with cognitive impairment.^22,26,27^ In a cross-sectional study with a sample of 92 residents in LTC, Allen et al. found that seniors who spent more time in verbal interaction with others were given more opioid medication (*r* = 0.22, *P* = 0.03).^26^ The contention that both nurses and physicians are reluctant to use opioids in LTC residents was supported, especially for those residents with cognitive impairment who were deemed nonpalliative.^22^ For example, a registered nurse said,^22^ “We tend to focus too much on pain control for palliation as opposed to just everyday clients … certainly nobody wants to die in pain but nobody wants to live in pain either.” Weissman and Matson found a widespread fear of treating pain without understanding the exact cause of pain, along with concern about overmedication and drug toxicity, especially for those seniors with cognitive impairment.^28^ Other barriers to effective pain management in LTC have been identified in the literature, including poor documentation, lack of interdisciplinary collaboration, poor nurse–physician communication, poor knowledge transfer, limited time, and resident and family knowledge and attitudes.^29–31^ The most commonly reported barrier is the lack of knowledge among care providers.^7–9,12,16,18,22^ Although a great deal of work has been conducted over the past couple of decades focusing on improving pain management in older adults, both nationally and internationally, pain practices illustrate that more work is still needed. In response to this need, a group of national leaders in pain management in older adults developed a national network of researchers and educators with the goal of establishing a national agenda to advance research and education for pain management in older adults.

Hence, the purpose of this study was to engage key stakeholders to develop a list of priority areas for future research and education to improve pain management in older adults. At the same time, we sought to identify barriers that needed to be addressed to meet these priority areas.

## Methods

A mixed methods design based on a modified Delphi approach was used for this study to promote group problem solving in an iterative process of problem definition, discussion, and feedback. Delphi methodology has been used previously to assist with priority setting in health care^32,33^ and is particularly appropriate when the face-to-face exchange of ideas is difficult and when scarcity of time and distances inhibit frequency of meetings.^34^ This study included three distinct components: a (1) qualitative component using focus groups with key informants or experts in the field of pain management in older adults, (2) review of the literature, and (3) survey of ranked responses completed by the same key informants who attended the focus groups. Each of these elements is described below.

### Qualitative component

We used purposive sampling with key informants from across Canada who were deemed experts in the field of pain management in older adults, based on record of publications and presentations on the topic of interest. We sought individuals from across Canada and from diverse backgrounds, (e.g., decision makers, researchers, health care workers, educators), disciplines (e.g., nurse, physician, psychologists, pharmacist, etc.), and settings of care (e.g., acute care, chronic pain clinic, long-term care, home care), including those who were members of the Canadian Pain Society with an interest in older people with pain. A formal letter was e-mailed to an initial list of 25 experts, requesting their participation in the study.

We held two focus groups with the key informants: one group who had expertise in pain education related to older adults (*n* = 8) and a second group with expertise in research about pain in older adults (*n* = 9). Both focus groups were facilitated by a trained moderator who guided the discussion (interview guide available upon request). Questions were asked about their perceptions of gaps in education or research (depending on their area of expertise) related to managing pain in older adults and barriers to addressing these gaps.

Data from the focus groups were recorded and analyzed using qualitative description methods. Important concepts that emerged from the data were labeled, categorized, and coded.^35,36^ Initial coding of each focus group was done independently by two individuals to foster credibility and dependability. Any discrepancies were reviewed by the investigators and discussed until consensus was reached.

### Scoping review of the literature

We conducted a scoping review using established methods^37,38^ to summarize the literature on priorities about education and research related to pain management in older adults from an international perspective to inform gaps in the existing research. Our goal was to explore and map all relevant literature on a broad topic and identify recurring themes, using rigorous and transparent methods to comprehensively search for all relevant literature and to analyze and interpret the data. As such, the criteria for exclusion and inclusion were not based on the quality of the studies but on relevance.

We searched Medline, CINAHL, Cochrane (including DARE), OVID SP, Web of Science, Ageline, and EMBASE using applicable Mesh headings and free text keywords (see Table 1). The journals yielding the greatest number of relevant articles—*Pain Research and Management, Pain Medicine*, and *Journal of Symptom and Pain Management*—were hand searched from January 2000 to January 2017.

**Table 1.** Keywords used in literature search.

Older adult	Pain management	Nursing home	Education
Over 65	Pain intervention	Community	Educational gaps
Aged	Pain assessment	Retirement home	Needs
Senior	Pain relief	Long-term care facility^a^	Priorities
Elderly	Pain medication	Long-term care home	Educate^a^
Resident		Long-term care setting	Guidelines
Older persons			Research
Geriatric			Systematic review
			Study
			Scoping review

**Table 2.** Ranked list of educational priorities related to pain management in older adults.

Educational priority	Ranked score (*n*)^a^
1. Effective and appropriate documentation of pain	23 (7)
2. Appropriate pain assessment strategies	17 (6)
3. Pain treatment for persons with dementia	17 (6)
4. Lack of postsecondary courses specific to pain management	17 (4)
5. Recognizing the complexities of pain	16 (5)
6. Lack of follow-up regarding efficacy of medication	14 (6)
7. Recognizing clinical signs of pain	14 (4)
8. Belief that it is normal for older adults to experience pain	11 (3)
9. Deficits in continued education	10 (3)
10. Lack of guidance regarding proper administration of pro re nata pain medications	10 (3)
11. Lack of funds to educate the public and create lobby groups	7 (3)
12. Fear of client overdose or adverse drug events	6 (2)
13. Pharmacological management of pain	5 (2)
14. Pharmacological treatments in relation to comorbidities	5 (2)
15. Including pain management and assessment under palliative care	4 (1)
16. Lack of public resources regarding pain education	2 (1)
17. Influence of traditions or beliefs on pain management	1 (1)
18. Psychosocial impacts of pain	1 (1)
19. Fear of repercussions from regulatory bodies	0 (0)
20. Lack of education resources available to the public	0 (0)
21. Role of nutrition in pain	0 (0)

^a^*n* indicates number of key informants who ranked item as one of their top 5 choices. Possible range of scores: 0–60.

**Table 3.** Ranked list of research priorities related to pain management in older adults.

Research priority	Ranked score (*n*)^a^
1. Understand the practice models of settings where pain management is successful	20 (7)
2. Costs of untreated pain in long-term care and other sectors	19 (6)
3. The effect of daily activities/mobility on pain management in long-term care residents	18 (6)
4. Understand patient preferences for pain management	17 (5)
5. Cost/benefit analysis of prevention vs. treatment of pain in older adults	15 (6)
6. Examine how to overcome research implementation barriers so that pain management strategies are sustainable	14 (5)
7. Cost/benefit of doing pain assessments	14 (4)
8. Compare the outcomes of pharmacological vs. nonpharmacological (e.g., behavioral therapy, exercise) interventions on pain management	13 (3)
9. Cost/benefit analysis of pharmacological vs. nonpharmacological interventions for pain management	12 (3)
10. Understand staff experiences working with older adults and pain management	9 (4)
11. Determine the effect of social engagement and recreational/leisure activities on pain management	8 (3)
12. Understand the factors that affect prescribers in accepting pain assessment information from the health care team	7 (6)
13. Understand what regulatory compliance items can be replaced with more effective pain management and other strategies in the LTC settings	7 (2)
14. Develop of pain management protocol	6 (2)
15. Understand the percentage of persons with undertreated pain on a regional basis	1 (1)
16. Assess the readiness of public stakeholder to advocate for pain management in older adults	0 (0)
17. Develop tools to assess quality of prescribing pain medications	0 (0)

^a^*n* indicates number of key informants out of possible 12 who ranked item as one of their top 5 choices. Possible range of scores: 0–60.

**Table 4.** Ranked list of barriers to address research or educational priority.

Barrier to address research or educational priority	Ranked score (*n*)^a^
1. Lack of staff time in performing pain assessments, treatments, evaluation (time to do the intervention)	22 (11)
2. Lack of resources to implement new pain management practices or interventions (e.g., staff time, change champion, dedicated time)	23 (7)
3. Unfamiliarity with tools designed to assess pain in seniors with dementia	24 (5)
4. Lack of communication and/or acceptance of pain assessment/pain management strategies between members of the interprofessional team	25 (4)
5. Difficulty of implementing research within current culture of care	26 (4)
6. Lack of continuity of care	27 (5)
7. Difficulty in changing current practices in community, hospital, and long-term care	28 (3)
8. Lack of time for proper pain management documentation	29 (4)
9. Lack of staff education on pain management	30 (3)
10. Lack of tools to assess the quality of prescribing pain medications	31 (0)
11. Lack of communication and/or acceptance of pain assessment and treatment information between personal support worker and registered staff in long-term care	32 (0)
12. Overcome the stigma of pain or opiophobia in older adults and their families	0 (0)

^a^*n* indicates number of key informants out of possible 12 who ranked item as one of their top 5 choices. Possible range of scores: 0–36.

Papers included in the synthesis were those that met the following criteria: all English-language papers including primary studies, literature and policy reviews, reports, editorials, essays, commentaries, and descriptive accounts published from January 2000 to January 2017.

Two members of the research team independently reviewed the abstracts and the articles using an iterative process of searching the literature, refining the search strategy, and reviewing articles for study inclusion.^37,38^ The following inclusion criteria were used: reference to educational or research issues or priorities related to older adults (defined as age 65 and older). Evidence above indicates that pain management is especially challenging when caring for older adults with cognitive impairments. However, we did not find it necessary to include cognitively impaired in our inclusion criteria because our goal was to capture the entire population of older adults.

Two members independently reviewed each paper and extracted data using Excel. We used a combination of descriptive tables, narrative syntheses,^39^ and team discussions during the data extraction process. To analyze the data extracted from the literature, we used a combination of tabular summaries and qualitative content analysis. The research team met to discuss the results of the aggregate data from each of the categories (priorities) within our data extraction tool.

**Figure 1.**
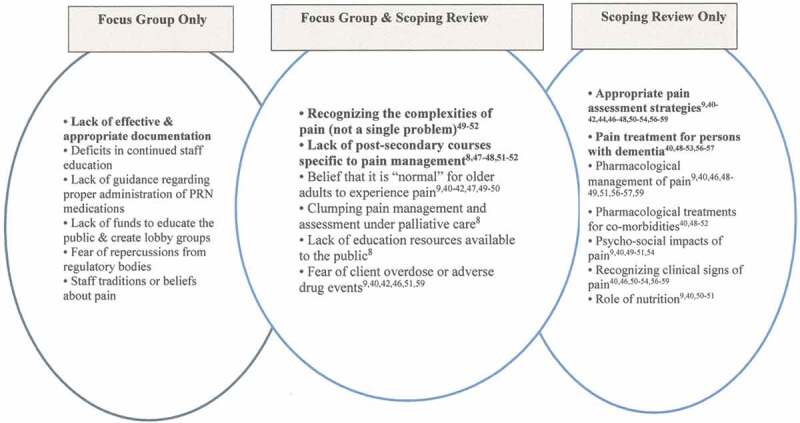
Educational priorities derived from focus groups and scoping review.

**Figure 2.**
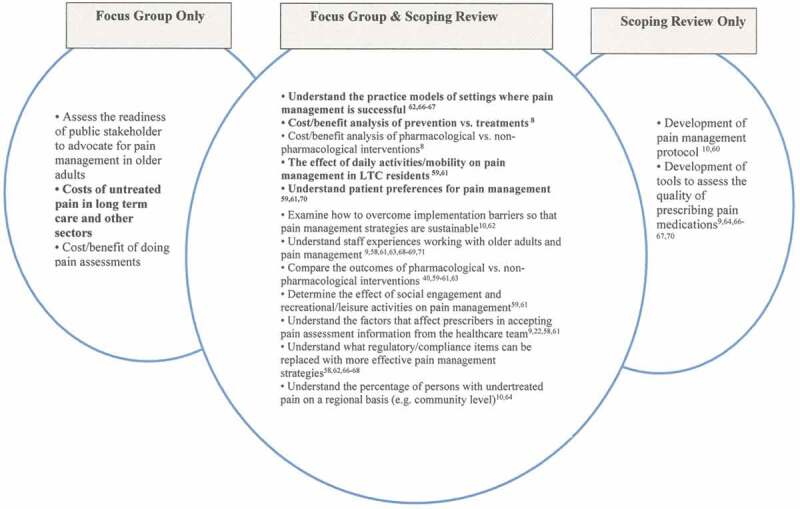
Research priorities derived from groups and scoping review.

Next, we compared focus group findings with findings from the scoping review to explore overlap between the two areas and where findings were identified in only one component (focus group vs. scoping review; see Figures 1 and 2). If a new item emerged from the scoping review that was not present in the focus group findings, we included it only if it was endorsed by three or more sources because our goal was to develop highly endorsed or key priorities, rather than a comprehensive list that has been developed previously.^40^

### Delphi survey

Based on the findings from the focus groups and scoping review, a comprehensive list was developed of the educational and research priorities identified within the context of the Canadian health care system. This list was input into an online survey and each key informant was sent an e-mail with a link to the survey and asked to complete it online. Of the priority lists for both education and research, key informants were asked to rank order their top five choices, with 1 indicating most important. For each barrier listed, key informants were asked to rank order their top three choices in the same manner. A reminder e-mail was sent two weeks after the original message to improve response rates.^33^

All completed surveys were analyzed using descriptive statistics. For each item, we weighted each ranking so that the highest rank, 1, received the highest score, 5. This scoring allowed us to calculate a ranked score for each research/education priority by summing each score from each key informant together. For each section (research and education priorities), scores had a possible range of 0–60, because there were 12 key informants and each item had a possible score of 0–5 (see Tables 2 and 3). Likewise, the barrier section had a possible range of 0–36, becausce each barrier had a possible score of 0–3 (see Table 4). We also calculated the frequency with which each item was endorsed by each of the key informants, with a possible range of 0–12 for each item.

## Results

### Characteristics of the sample

**Figure 3.**
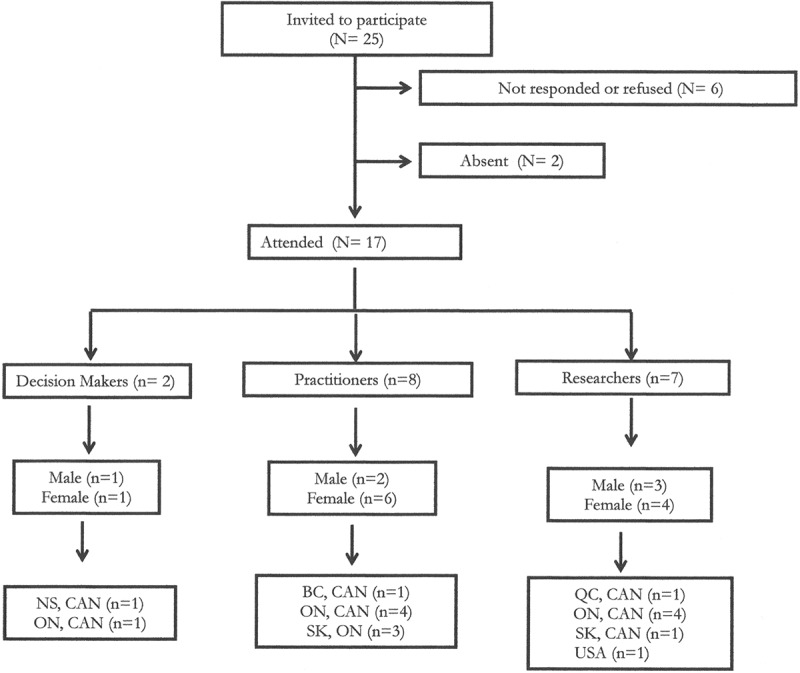
Description of focus group participants.

Of those invited, 17 key informants participated in a focus group for a response rate of 68% (see Figure 3). The majority of participants were female (*n* = 11; 65%). Almost half of the participants were practitioners (*n* = 8; 47%), including nurses, physicians, psychologists, pharmacists; 41% (*n* = 7) were researchers; and 12% (*n* = 2) were decision makers, including a clinical manager and director of a professional organization. There was representation from across Canada; 53% (*n* = 9) from Ontario, 24% (*n* = 4) from Saskatchewan, and 6% (*n* = 1) from each of British Columbia, Quebec, and Nova Scotia. One other participant was from the United States.

### Focus groups

Twenty-nine priority issues were identified through the focus groups, 13 related to education and 15 related to research. The education priorities aligned with seven main categories: (1) addressing education deficits (at both the prelicensure and continuing education levels); (2) recognizing gaps or areas that need improvement in appropriate medication administration, documentation, and follow-up; (3) acknowledging the fear factor regarding opioid use; (4) addressing false beliefs and misconceptions regarding pain in older adults; (5) recognizing differences in cultural practices; (6) addressing gaps within the interprofessional team; and (7) raising public awareness regarding the impact of pain on older adults.

The research priorities focused on four key areas: (1) understanding the current context of care and patient preferences in LTC to help develop successful pain management interventions; (2) examining nonpharmacological interventions (e.g., exercise, behavioral therapy, social engagement, leisure activities) to improve pain and quality of life; (3) examining costs versus benefits of pain assessment and management approaches; and (4) exploring system-level issues that impact pain management at the resident level.

In terms of the barriers that need to be addressed to meet these priorities, the majority of discussion focused on the lack of resources, time, and staff to fully implement pain management practices effectively. Other barriers included poor communication among staff members and lack of knowledge for staff about pain management.

### Scoping review

**Figure 4.**
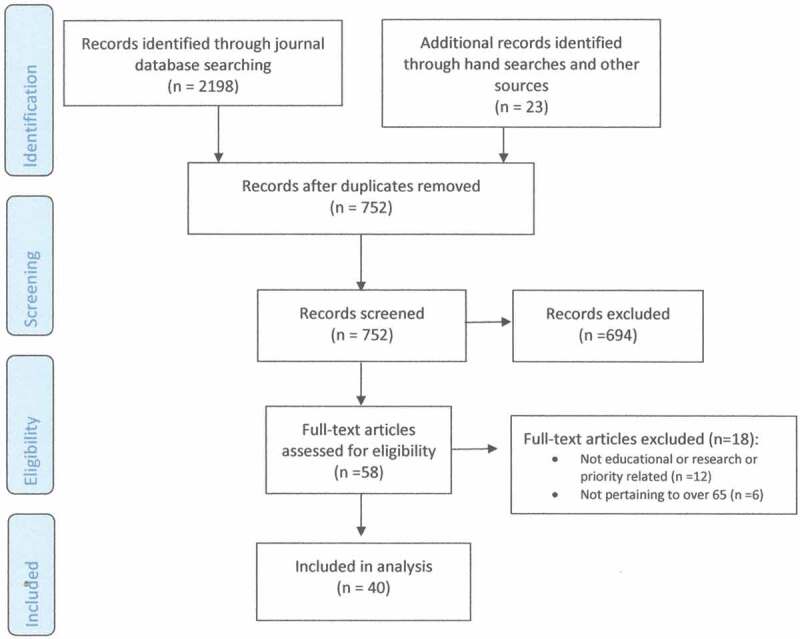
Flowchart illustrating search and selection.

An initial search through databases and grey literature yielded a total of 752 results. A PRISMA flowchart demonstrating the search and study process is presented in Figure 4. Literature was screened using the inclusion and exclusion criteria outlined above, which resulted in 694 papers being excluded; 292 papers were not educational or research or priority related and 402 papers did not pertain to older adults over 65. The full text of the remaining 58 papers were reviewed and assessed for eligibility and 40 papers were included in the final analysis.

The scoping review validated seven out of 13 of the education priorities that were identified through the focus groups, as well as added seven more key priorities to our list (see Figure 1). The most highly endorsed education priorities were (1) the belief that it is normal for older adults to experience pain,^9,40–45^ (2) appropriate pain assessment strategies,^40–43,45,47–53,55–58^ (3) pharmacological management of pain,^9,40,44,48–50,54–56,58^ (4) fear of client overdose or adverse drug events,^9,40,42,48,50,58^ (5) pain treatment for persons with dementia,^40,44,45,50–53,55,56^ (6) lack of postsecondary courses specific to pain management,^40,43,49–51^ and (7) recognizing clinical signs of pain.^40,45,48,50–53,55–58^

For research priorities, 12 out of 15 of the priorities identified through the focus groups were supported by the scoping review results with the scoping review adding another twp priorities to the list (see Figure 2). The most highly endorsed priorities were the need to (1) understand staff experiences working with older adults and pain management,^9,57,59–63^ (2) understand what regulatory/compliance items can be replaced with more effective pain management or other strategies in LTC,^9,57,59–61,63^ (3) compare outcomes of pharmacological versus nonpharmacological interventions on pain management,^40,58–60,64^ (4) understand the factors that affect prescribers in accepting pain assessment information from the health care team,^9,22,57,59^ and (5) develop tools to assess the quality of prescribing pain medications.^7,9,65–67^

### Delphi survey

The Delphi survey was completed by 71% (*n* = 12) of the focus group participants. Based on their responses, the following ranked education priorities received the highest endorsement: (1) effective and appropriate documentation of pain, (2) appropriate pain assessment strategies, (3) pain treatment for persons with dementia, (4) lack of postsecondary courses (at both the prelicensure and continuing education levels) specific to pain management, and (5) recognizing the complexities of pain. In terms of research priorities, the following five priorities received the highest ranked score: (1) understanding the practice models of settings where pain management is successful, (2) exploring the costs of untreated pain in LTC and other sectors, (3) examining the effect of daily activities/mobility on pain management in LTC residents, (4) understanding patient preferences for pain management, and (5) analyzing the costs/benefits of prevention versus treatment of pain in older adults. The top three barriers that were reported as impediments to these priorities were (1) lack of staff time in performing pain assessments, treatments, evaluation (time to do the intervention); (2) lack of resources to implement new pain management practices or interventions (e.g., change champion, dedicated time); and (3) unfamiliarity with tools designed to assess pain in older adults with dementia.

## Discussion

These study findings highlight key priorities and related barriers to improving pain management in older adults in the areas of research and education that have been identified using a systematic approach. These findings can provide direction for future work and contribute to the development of a national agenda of key priorities in this field.

These findings are consistent with previous work in this area, reporting that key educational needs related to pain management in older adults include pain assessment strategies, pharmacological and nonpharmacological treatments, and how to distinguish pain expression from other behaviors that are commonly observed in this population, particularly those who have dementia.^40^ Key barriers to addressing these priorities include lack of staff time and resources available to them. These findings are consistent with the work of Stolee et al., who identified a list of factors in LTC hospitals that impact the effectiveness of continuing education programs, including a changing resident population, staff resistance to change, workforce educational background, management support, and available resources.^68^ Future work is needed to address these key priority areas while being mindful of the barriers and constraints that impede successful changes in practice.

Our study identified lack of effective and appropriate documentation of pain as another key priority, which is paramount and often perceived to be a fairly straightforward intervention to implement. However, the barriers that were identified to addressing this priority emphasize the complexity in changing practice, which relies on effective interdisciplinary communication to ensure timely follow-up with pain treatments to assess their effectiveness. Hence, a single education session is not likely to improve practice, which is well supported in the literature.^69,70^ On the other hand, more complex interventions appear to have an impact on improving pain management for older adults. For example, Long found that after an intensive training program and onsite consultation with the concomitant changes in policies, procedures, and documentation, professional and staff knowledge improved after 6 months, attitudes changed, and barriers were mitigated.^71^ With a comprehensive quality improvement pain plan in place, the findings suggest that education in pain management in long-term care and program changes that adopt best practices in pain can make a difference.

Clearly, research needs to focus on ways to address efficient assessment practices and interventions that staff are able to implement in timely manner, as well as strategies to educate staff that create less burden on staff time, given the lack of time and competing demands that LTC homes are currently facing. Wagner et al. suggest the use of interdisciplinary “huddles,” which enable teams to have short but frequent briefings, offering a mechanism for immediate learning in LTC homes.^72^ Evidence on the use of huddles in acute care shows that workplace culture, communication, collaboration, and staff satisfaction improves.^73^

Finally, this study methodology provided a way to examine key priorities from an international perspective (scoping review), followed by the identification of additional priorities (focus groups) and overall ranking of these priorities (Delphi) with Canadian pain experts in the field of aging. In this manner, the priority setting of research and education priorities is ultimately grounded within the Canadian context. Given the emphasis on optimizing health care costs in the Canadian system, it is not surprising that two of our top research priorities focus on exploring costs of untreated pain or pain prevention. Costs related to pain management are also recognized as a priority in strategic initiatives in the United States,^74^ Australia,^75^ Norway,^76^ Portugal,^77^ and Wales.^78^ The National Pain Awareness Campaign, led by the Canadian Pain Society (see 79), has likely influenced our study findings because the need to educate the public and create lobby groups was identified as a priority within the focus groups but not the internationally based scoping review. Moreover, the emphasis in Canada on improving pain education,^79^ at both the postsecondary (e.g., college, university) and continuing education levels was highlighted in our findings as a key priority. Lastly, the heavy regulation of long-term care homes in Canada was raised as a concern and related research priority in both the Canadian and U.S. literature.^57,61,66,67,80^

Although this study used a small sample, triangulation of methods helped improve rigor. Akins et al. examined what constituted a sufficient number of Delphi survey participants to ensure stability of results.^81^ Results from their study indicate that the response characteristics of a small expert panel in a well-defined area are stable in light of augmented sampling.^79^ Another limitation to this study could be its lack of generalizability to countries other than Canada because the sample used included only pain experts within Canada. Although the initial purpose of this initiative was to establish education and research priorities for Canada, the scoping review drew from global literature that identified priorities from other countries as well, thus adding merit to establishing these priorities for other countries as well. The ordering of those priorities based on input from key informants is Canadian, but the list of priorities that came from scoping review applies more broadly.

It is our intent that this study will advance education and research in the area of pain management in older adults forward by putting forward a call to action for government, educators, policymakers, and funders to dedicate resource to address priorities. The findings from this study argue for educational organizations that train health care providers in LTC to prioritize pain content in the curriculum specifically focused on pain and aging issues. In addition, there is a need for targeted funding calls to address pain and aging research priorities.

## Conclusion

These findings highlight priority issues related to pain management in older adults from a nationwide perspective. This work can provide a basis on which to further develop curricula at both the undergraduate and continuing education levels as well as provide a basis for funding agencies and researchers alike to enrich the research agenda across Canada. In doing so, it is hoped that pain management is improved for older adults and unnecessary suffering is alleviated.

## References

[1] Statistics Canada. Population projections for Canada, provinces and territories 2009 to 2036. Ottawa (Canada): Minister of Industry; 2010.

[2] Rice AS, Smith B,H, Blyth F,M. Pain and the gobal burden of disease. Int Assoc Study Pain. 2016;157(4):791–796. 10.1097/j.pain.0000000000000454 26670465

[3] Patel K, Guralnik J, Dansie E, Turk D. Prevalence and impact of pain among older adults in the United States: findings from the 2011 National Health and Aging Trends Study. Pain. 2013;154(12):2649–2657. doi:10.1016/j.pain.2013.07.029. 24287107 PMC3843850

[4] Lapane KL, Quilliam BJ, Chow W, Kim MS. Impact of revisions to the F-Tag 309 surveyors’ interpretive guidelines on pain management among nursing home residents. Drugs Aging. 2012;29(5):385–393. doi:10.2165/11599340-000000000-00000. 22462594

[5] Gianni W, Madaio RA, Di Cioccio L, D’Amico F, Policicchio D, Postacchini D, Franchi F, Ceci M, Benincasa E, Gentili M, et al. Prevalence of pain in elderly hospitalized patients. Arch Gerontol Geriatr. 2010;51(3):273–276. doi:10.1016/j.archger.2009.11.016. 20031238

[6] Eggermont L, Leveille S, Shi L, Kiely D, Shmerling R, Jones R, Guralnik J, Bean J. Pain characteristics associated with the onset of disability in older adults: the maintenance of balance, independent living, intellect, and zest in the Elderly Boston Study. J Am Geriatr Soc. 2014;62(6):1007–1016. doi:10.1111/jgs.2014.62.issue-6. 24823985 PMC4057984

[7] Herr K, Titler M, Fine P, Sanders S, Cananaugh J, Swegle J, Tang X, Forcucci C. The effect of a translating research into practice (TRIP)—cancer intervention on cancer pain management in older adults in hospice. Pain Med. 2012;13(8):1004–1017. doi:10.1111/j.1526-4637.2012.01405.x. 22758921 PMC3422373

[8] Smith A, Murray B. Managing pain in older adults. J Pain. 2012;1(1):1–2.

[9] Auret K, Schug A. Underutilisation of opioids in elderly patients with chronic pain: approaches to correcting the problem. Drugs Aging. 2005;22(8):641–654. doi:10.2165/00002512-200522080-00002. 16060715

[10] Allcock N, McGarry J, Elkan R. Management of pain in older people within the nursing home: a preliminary study. Health Soc Care Community. 2002;10(6):464–471. doi:10.1046/j.1365-2524.2002.00392.x. 12485133

[11] Zwakhalen SMG, Hamers JPH, Abu-Saad HH, Berger MPF. Pain in elderly people with severe dementia: a systematic review of behavioural pain assessment tools. BMC Geriatr. 2006;6(1):1471–2318. doi:10.1186/1471-2318-6-3. PMC1397844 16441889

[12] Husebo BS, Achterberg WP, Lobbezoo F, Kunz M, Lautenbacher S, Kappesser J, Tudose C, Inger Strand L. Pain in patients with dementia: a review of pain assessment and treatment challenges. Nor Epidemiol. 2012;22(2):243–251.

[13] Blyth FM, Cumming R, Mitchell P, Wang JJ. Pain and falls in older people. Eur J Pain. 2007;11(5):564–571. doi:10.1016/j.ejpain.2006.08.001. 17015026

[14] Hadjistavropoulos T, Hunter P, Dever Fitzgerald T. Pain assessment and management in older adults: conceptual issues and clinical challenges. Can Psychol. 2009;50:241–254. doi:10.1037/a0015341.

[15] Hadjistavropoulos T, Herr K, Prkachin K, Craig K, Gibson S, Lukas L, Smith J. Pain assessment in elderly adults with dementia. Lancet Neurol. 2014;13:1216–1227. doi:10.1016/S1474-4422(14)70103-6. 25453461

[16] Horgas A, Tsai P. Analgesic drug prescription and use in cognitively impaired nursing home residents. Nurs Res. 1998;47(4):235–242. doi:10.1097/00006199-199807000-00009. 9683119

[17] Mezinskis PM, Keller AW, Luggen AS. Assessment of pain in the cognitively impaired older adult in long-term care. Geriatr Nurs. 2004;25(2):107–112. doi:10.1016/j.gerinurse.2003.09.005. 15107794

[18] McAuliffe L, Brown D, Fetherstonhaugh D. Pain and dementia: an overview of the literature. Int J Older People Nurs. 2012;7(3):219–226. doi:10.1111/opn.2012.7.issue-3. 22830419

[19] Takai Y, Yamanoto-Mitani N, Okamoto Y, Koyama K, Honda A. Literature review of pain prevalence among older residents of nursing homes. Pain Manag Nurs. 2010;11(4):209–223. doi:10.1016/j.pmn.2010.08.006. 21095596

[20] Herr K, Bjoro K, Decker S. Tools for assessment of pain in nonverbal older adults with dementia: a state-of-the-science review. J Pain Symptom Manage. 2006;31:170–192. doi:10.1016/j.jpainsymman.2005.07.001. 16488350

[21] Chibnall J, Tait R. Pain assessment in cognitively impaired and unimpaired older adults: a comparison of four scales. Pain. 2001;91(1–2):173–186. doi:10.1016/S0304-3959(00)00485-1. 11323138

[22] Kaasalainen S, Coker E, Dolovich L, Papaioannou A, Hadjistavropoulos T, Emili A, Ploeg J. Pain management decision-making among long-term care physicians and nurses. West J Nurs Res. 2007;29(5):561–580. doi:10.1177/0193945906295522. 17548894 PMC5104556

[23] Martin R, Williams J, Hadjistavropoulos T, Hadjistavropoulos H, MacLean M. A qualitative investigation of seniors’ and caregivers’ views on pain assessment and management. Can J Nurs Res. 2005;37(2):142–165. 16092785

[24] Aubin M, Giguere A, Hadjistavropoulos T, Verreault R. The systematic evaluation of instruments designed to assess pain in persons with limited ability to communicate. Pain Res Manag. 2007;12(3):195–203. doi:10.1155/2007/705616. 17717611 PMC2670710

[25] Lichtner V, Dowding D, Esterhuizen P, Closs J, Long A, Corbett A, Briggs M. Pain assessment for people with dementia: a systematic review of systematic reviews of pain assessment tools. BMC Geriatrics. 2014;14:138:1–15. doi:10.1186/1471-2318-14-138. 25519741 PMC4289543

[26] Allen R, Thorn B, Fisher S, Gerstle J, Quarles K, Bourgeois M, Dijkstra K, Burgio L. Prescription and dosage of analgesic medication in relation to resident bebaviors in the nursing home. J Am Geriatr Soc. 2003;51(4):534–538. doi:10.1046/j.1532-5415.2003.51164.x. 12657075 PMC2670933

[27] Morrison R, Siu A. A comparison of pain and its treatment in advanced dementia and cognitively intact patients with hip fractures. J Pain Symptom Manag. 2000;19(4):240–248. doi:10.1016/S0885-3924(00)00113-5. 10799790

[28] Weissman D, Matson S. Pain assessment and management in the long-term care setting. Theor Med. 1999;20:31–43. doi:10.1023/A:1009923907285. 10442052

[29] Tarzian A, Hoffman D. Barriers to managing pain in the nursing home: findings for a statewide survey. J Am Med Dir Assoc. 2004;5:82–88. 14984618 10.1097/01.JAM.0000110648.46882.B3

[30] Stevenson K, Dahl J, Berry P, Beck S, Griffie J. Institutionalizing effective pain management practices: practice change programs to improve the quality of pain management in small health care organizations. J Pain Symptom Manag. 2006;31(3):248–261. doi:10.1016/j.jpainsymman.2005.07.002. 16563319

[31] Jones K, Fink R, Pepper G, Hutt E, Vojir C, Scott J, Clark L, Mellis K. Improving nursing home staff knowledge and attitudes about pain. Gerontologist. 2004;44(4):469–478. doi:10.1093/geront/44.4.469. 15331804

[32] Burnette D, Morrow-Howell N, Chen LM. Setting priorities for gerontological social work research: a national Delphi study. Gerontologist. 2003;43(6):828–838. doi:10.1093/geront/43.6.828. 14704382

[33] Moscovice IP, Armstrong P, Shortell S, Bennett R. Health services research for decision-makers: the use of the Delphi technique to determine health priorities. J Health Polit Policy Law. 1977;2(3):388–410. doi:10.1215/03616878-2-3-388. 591704

[34] McBride AJ, Pates R, Ramadan R, McGowan C. Delphi survey of experts’ opinions on strategies used by community pharmacists to reduce over-the-counter drug misuse. Addiction. 2003;98(4):487–497. doi:10.1046/j.1360-0443.2003.00345.x. 12653818

[35] Patton MQ. Qualitative research & evaluation methods. 3rd ed. Thousand Oaks (CA): Sage; 2002.

[36] Sandelowski M. Whatever happened to qualitative description? Res Nurs Health. 2000;23:334–340. doi:10.1002/1098-240X(200008)23:4<334::AID-NUR9>3.0.CO;2-G. 10940958

[37] Arskey H, O’Malley L. Scoping studies: towards a methodological framework. Int J Soc Res Methodol. 2005;8(1):19–32. doi:10.1080/1364557032000119616.

[38] Levac D, Colquhoun H, O’Brien KK. Scoping studies: advancing the methodology. Implement Sci. 2010;5(69):1–9. doi:10.1186/1748-5908-5-69. 20854677 PMC2954944

[39] Mays N, Pope C, Popay J. Systematically reviewing qualitative and quantitative evidence to inform management and policy-making in the health field. J Health Serv Res Policy. 2005;10(Suppl. 1):6–20. doi:10.1258/1355819054308576. 16053580

[40] Tousignant-Laflamme Y, Tousignant M, Lussier D, Lebel P, Savoie M, Lalonde L, Choinière M. Educational needs of health care providers working in long-term care facilities with regard to pain management. Pain Res Manag. 2012;17(5):341–346. 10.1155/2012/506352 PMC3465095 23061085

[41] Schofield P. Pain education and current curricula for older adults. Pain Med. 2012;13:S51–S56. doi:10.1111/j.1526-4637.2011.01283.x. 22497748

[42] Vallerand AH, Hasenau SM, Templin T. Barriers to pain management by home care nurses. Home Healthc Nurse. 2004;22(12):831–838. 15597004

[43] Schofield P, Sofaer-Bennett B, Hadjistavropolous T, Zwakhalen S, Brown C, Westerling D, Wright S. A collaborative eXpert literature review of pain education, assessment and management. Aging Health. 2012;8(1):43–54. doi:10.2217/ahe.11.53.

[44] Reid M, Bennett D, Chen W, Eldadah B, Farrar J, Ferrell B, Gallagher RM, Hanlon JT, Herr K, Horn SD, et al. Improving the pharmacological management of pain in older adults: identifying the research gaps and methods to address them. Pain Med. 2011;12:1336–1357. doi:10.1111/j.1526-4637.2011.01211.x. 21834914 PMC3173592

[45] Herr K. Pain assessment strategies in older adults. J Pain. 2010;12(3):3–13. doi:10.1016/j.jpain.2010.11.011. 21396599

[46] Loman R, Ahern M, Wright A, Brown L. Nurses’ knowledge of pain in the elderly. J Pain Symptom Manage. 2001;21(4):317–322. doi:10.1016/S0885-3924(01)00248-2. 11312046

[47] Green CR, Wheeler JR, Marchant B, LaPorte F, Guerrero E. Analysis of the physician variable in pain management. Pain Med. 2001;2(4):317–327. doi:10.1046/j.1526-4637.2001.01045.x. 15102236

[48] Twycross A. Educating nurses about pain management: the way forward. J Clin Nurs. 2002;11(6):705–714. doi:10.1046/j.1365-2702.2002.00677.x. 12427174

[49] Murinson B, Gordin V, Flynn S, Driver L, Gallagher R, Grabois M. Recommendations for a new curriculum in pain medicine for medical students: toward a career distinguished by competence and compassion. Pain Med. 2013;14:345–350. doi:10.1111/pme.12051. 23387441 PMC6069634

[50] Turner G, Weiner D. Essential components of a medical student curriculum on chronic pain management in older adults: results of a modified delphi process. Pain Med. 2002;3(3):240–252. doi:10.1046/j.1526-4637.2002.02030.x. 15099259

[51] Kovach CR, Griffie J, Muchka S, Noonan PE, Weissman DE. Nurses’ perceptions of pain assessment and treatment in the cognitively impaired elderly: it’s not a guessing game. Clin Nurse Spec. 2000;14(5):215–220. doi:10.1097/00002800-200009000-00011. 11188470

[52] Monroe TB, Parish A, Mion LC. Decision factors nurses use to assess pain in nursing home residents with dementia. Arch Psychiatr Nurs. 2015;29(5):316–320. doi:10.1016/j.apnu.2015.05.007. 26397435 PMC4580913

[53] Becker G, Momm F, Deibert P, Xander C, Gigle A, Wagner B, Baumgartner J. Planning training seminars in palliative care: a cross-sectional survey on the preferences of general practitioners and nurses in Austria. Biomed Med Educ. 2010;10(43):1–7. 10.1186/1472-6920-10-43 PMC2893516 20540757

[54] Cohen-Mansfield J. Nursing staff members’ assessments of pain in cognitively impaired nursing home residents. Pain Manag Nurs. 2005;6(2). doi:10.1016/j.pmn.2005.05.002. 15970920

[55] Chandler R, Bruneau B. Barriers to the management of pain in dementia care. Nursing Practice Review. 2015;110(28). 25087440

[56] Gilmore-Bykovskyi AL, Bowers BJ. Understanding nurses’ decisions to treat pain in nursing home residents with dementia. Res Gereontol Nurs. 2013;6(2):127–138. 10.3928/19404921-20130110-02 PMC3634876 23330944

[57] Hadjistavropoulos T, Kaasalainen S, Williams J, Zacharias R. Improving pain assessment practices and outcomes in long-term care facilities: a mixed methods investigation. Pain Manag Nurs. 2014;15(4):748–759. doi:10.1016/j.pmn.2013.07.009. 24157227

[58] Blomqvist K, Edberg AK. Living with persistent pain: experiences of older people receiving home care. J Adv Nurs. 2002;40(3):297–306. doi:10.1046/j.1365-2648.2002.02371.x. 12383181

[59] Martin R, Williams J, Hadjistavropoulos T, Hadjistavropoulos HD, MacLean M. A qualitative investigation of seniors’ and caregivers’ views on pain assessment and management. Can J Nurs Res. 2005;37(2):142–165. 16092785

[60] Manias E. Complexities of pain assessment and management in hospitalised older people: a qualitative observation and interview study. Int J Nurs Stud. 2012;49:1243–1254. doi:10.1016/j.ijnurstu.2012.05.002. 22640777

[61] Keeney CE, Scharfenberger JA, O’Brien JG, Looney S, Pfeifer MP, Hermann CP. Initiating and sustaining a standardized pain management program in long-term care facilities. J Am Med Dir Assoc. 2008;9(5):347–353. doi:10.1016/j.jamda.2008.02.004. 18519117

[62] Cadogan MP, Schnelle JF, Al-Sammarrai M, Yamamoto-Mitani N, Cabrera G, Osterweil D, Simmons SF. A standardized quality assessment system to evaluate pain detection and management in the nursing home. J Am Med Dir Assoc. 2005;6:1–9. doi:10.1016/j.jamda.2004.12.002. 15871864

[63] Kaasalainen S, Brazil K, Coker E, Ploeg J, Martin-Misener R, Donald F, DiCenso A, Hadjistavropoulos T, Dolovich L, Papaioannou A, et al. An action-based approach to improving pain management in long-term care. Can J Aging. 2010;29(4):503–517. doi:10.1017/S0714980810000528. 21134301

[64] Horgas AL. Pain management in elderly adults. J Infus Nurs. 2003;26(3):161–165. doi:10.1097/00129804-200305000-00007. 12792374

[65] Hutt E, Pepper GA, Vojir C, Fink R, Jones KR. Assessing the appropriateness of pain medication prescribing practices in nursing homes. J Am Geriatr Soc. 2006;54(2):231–239. doi:10.1111/jgs.2006.54.issue-2. 16460373

[66] Herr KA, Garand L. Assessment and measurement of pain in older adults. Clin Geriatr Med. 2001;17(3):457–478. doi:10.1016/S0749-0690(05)70080-X. 11459715 PMC3097898

[67] Herr K. Pain in the older adult: an imperative across all health care settings. Pain Manag Nurs. 2010;11(2):S1–S10. doi:10.1016/j.pmn.2010.03.005. 20510844

[68] Stolee P, Esbaugh J, Aylward S, Cathers T, Harvey DP, Hillier LM, Keat N, Feightener JW. Factors associated with the effectiveness of continuing education in long-term care. Gerontologist. 2005;45(3):399–405. doi:10.1093/geront/45.3.399. 15933280

[69] Hasson H. Systematic evaluation of implementation fidelity of complex interventions in health and social care. Implement Sci. 2010;5:67–76. doi:10.1186/1748-5908-5-67. 20815872 PMC2942793

[70] Stevenson KM, Dahl JL, Berry PH, Beck SL, Griffie J. Institutionalizing effective pain management practices: practice change programs to improve the quality of pain management in small health care organizations. J Pain Symptom Manage. 2006;31:248–261. doi:10.1016/j.jpainsymman.2005.07.002. 16563319

[71] Long C. Pain management education in long-term care: it can make a difference. Pain Manag Nurs. 2013;14(4):220–227. doi:10.1016/j.pmn.2011.04.005. 24315245

[72] Wagner LM, Huijbregts M, Sokoloff LG, Wisniewski R, Walsh L, Feldman S, Conn DK. Implementation of mental health huddles on dementia care units. Can J Aging. 2014;33(3):235–245. doi:10.1017/S0714980814000166. 26261887

[73] Quigley P, White S. Hospital-based fall program measurement and improvement in high reliability organizations. Online J Issues Nurs. 2013;18(2). 23758423

[74] Department of Health and Human Services. National pain strategy: a comprehensive population health-level strategy for pain; 2016 [accessed October 17, 2017]. https://iprcc.nih.gov/docs/DraftHHSNationalPainStrategy.pdf.

[75] National Pain Summit Initiative. National pain strategy: pain management for all Australians. Melbourne (Australia): Australian Pain Society. 2010 [accessed October 17, 2017]. https://www.iasp-pain.org/files/Content/NavigationMenu/Advocacy/InternationalPainSummit/Australia_2010PainStrategy.pdf.

[76] Hara KW, Borchgrevink P. National guidelines for evaluating pain—patients’ legal right to prioritised health care at multidisciplinary pain clinics in Norway implemented 2009. Scand J Pain. 2010;1:60–63. doi:10.1016/j.sjpain.2009.10.002. 29913923

[77] Directorate-General for Health. National program for pain management [accessed October 17, 2017]. https://www.iasp-pain.org/files/Content/NavigationMenu/Advocacy/InternationalPainSummit/PortugalPainStrategy.pdf.

[78] Welsh Government: Health and Social Services. Service development and commissioning services designed for people with chronic conditions. Llywodraeth Cynulliad Cymru Welsh Assembly Government. 2009 [accessed October 17, 2017]. https://www.iasp-pain.org/files/Content/NavigationMenu/Advocacy/InternationalPainSummit/WalesDirectives.pdf.

[79] The Canadian Pain Society CPS position statement; 2010 [accessed 2017 July 22]. http://www.canadianpainsociety.ca/?page=PositionStatement.

[80] Kaasalainen S, Brazil K, Akhtar-Danesh N, Coker E, Ploeg J, Donald F, Martin-Misener R, DiCenso A, Hadjistavropoulos T, Dolovich L, Papaioannou A. The evaluation of an interdisciplinary pain protocol in long-term care. J Am Med Dir Assoc. 2012;13(7):664–668. doi:10.1016/j.jamda.2012.05.013. 22739020

[81] Akins RB, Tolson H, Cole BR. Stability of response characteristics of a Delphi panel: application of bootstrap data expansion. BMC Med Res Methodol. 2005;5(37):1–12. doi:10.1186/1471-2288-5-37. 16321161 PMC1318466

